# Supplementary material for: Hydroxyl-rich macromolecules enable the bio-inspired synthesis of single crystal nanocomposites
Source: Nat Commun. 2019 Dec 12;10:5682. doi: 10.1038/s41467-019-13422-9 (PMC6908585; doi:10.1038/s41467-019-13422-9)
Supplement: Supplementary file 1 — Supplementary Information [file 41467_2019_13422_MOESM1_ESM.pdf]

Supplementary Information

Hydroxyl-Rich Macromolecules Enable the Bio-Inspired Synthesis of Single Crystal Nanocomposites

Yi-Yeoun Kim, Robert Darkins, Alexander Broad, Alexander N. Kulak, Mark A. Holden, Ouassef Nahi, Steven P. Armes, Chiu C. Tang, Rebecca F. Thompson, Frederic Marin, Dorothy M. Duffy, and Fiona C. Meldrum

Contents:

- **Supplementary notes 1-3**
- **Supplementary figures 1-12**
- **Supplementary tables 1-4**
- **Supplementary video legends 1-4**
- **Supplementary references (1-3)**

Supplementary Note 1. Estimation of Inter-particle Distance of PGMA-NPs in Calcite Crystals.

The volume fraction of the NPs within the calcite crystals was estimated using a weight-based method (AAS and XRD) and was then converted to a volume percentage of Au NPs. The volume percentage of Au NPs was also directly measured from the electron tomography images, where a threshold contrast level was set to determine the boundaries of each particle. The total void volume in the sampled volume was then calculated by approximating the NPs to spheres with average diameters of 4 or 14 nm, as determined from the TEM analysis of the NPs. The obtained volume fraction was higher than the value obtained from AA and XRD analysis. Based on the obtained volume fraction, the mean inter-particle distance was calculated using equation (1), where D is particle diameter and f is volume fraction.

$$interparticle\ distance\ x = D\left[\sqrt[3]{\frac{0.64}{f}} - 1\right] \qquad \text{Equation (1)}$$

Supplementary Note 2: Plasmon Coupling in PGMA-NPs/Calcite Nanocomposite Crystals.

The extinction of the gold NPs in water (refractive index 1.33) was measured as 526 nm, and the extinction efficiency of the gold NPs in calcite (refractive index 1.66) was estimated as 555 nm using Mie scattering theory.[1] The influence of plasmon coupling on the absorption spectrum of Au NPs in calcite was then estimated using the universal ruler equation (equation 2):[2]

$$\frac{\Delta\lambda}{\lambda} = ae^{-(\frac{T}{d}-0.414)/\tau} \qquad \text{Equation (2)}$$

where Δλ is wavelength shift, λ is the extinction wavelength of Au nanoparticles in water, τ is the decay constant, a is the particle coupling strength, T is the inter-particle separation (edge-to-edge distance) and d is the particle diameter. A decay constant of τ = 0.3-0.37 and particle coupling strength of *a* = 0.2-0.3 provided a good fit to our experimental results. The estimated wavelength versus gap distance is plotted using universal ruler equation in Supplementary Figure 11a and UV-Vis spectra of protein- and polymer-stabilised NPs in Supplementary Figure 11b.

Supplementary Note 3: In-situ AFM Analysis of Particle Occlusion in Calcite.

The incorporation of PGMA-NPs in calcite is shown in Figure 5. We further investigated nanoparticle incorporation in calcite using alpha-1-glycoprotein NPs (GP-NPs). The micrographs in Supplementary Figure 12 show GP-NPs absorbed on the calcite surface and incorporated into the crystal. The acute and obtuse step morphologies are not significantly affected by the presence of the GP-NPs, which is consistent with the macroscopic observations that show no distortions from typical calcite rhombohedral morphologies. For GP-NPs, even at low saturations, there appears to be no strong preference for acute or obtuse steps, in contrast to the PGMA-NPs, which preferentially associated with acute steps. In common with the PGMA-NPs, the GP-NPs are found preferentially at defects on the surface, such as cracks. Supplementary Figure 12 shows one such example, where several GP-NPs are incorporated over several minutes at a crack on the crystal surface.

The experiments shown in Figure 5 and Supplementary Figure 12 were obtained using Peak Force Tapping mode. This mode allows imaging of the growing calcite surface at lower contact forces (<1 nN). The exact forces applied varied throughout the experiment, and were optimised to reduce the tip-sample interactions as far as possible, whilst still resolving the growing crystal surface. Reducing tip-sample interactions is crucial in these experiments, where relatively weak binding additives are otherwise displaced by the AFM tip during scanning. In both contact mode (with contact forces minimised) and tapping mode, the gold particles were removed from the scan area. This type of tip-sample interaction has been previously observed in AFM measurements of particles on surfaces.[3]

We illustrate the potential issues from tip-sample interactions in Supplementary Figure 8. The two images In Supplementary Figure 8a-b show consecutive AFM images obtained in the same area at different magnifications of the PGMA-NPs system. The scan area in the higher magnification image is observed clearly in the lower magnification image, which shows that the tip has displaced a significant proportion of the particles in this area during scanning. Those particles that were not displaced may

have been partially incorporated, at which point the same particle can be imaged over several scans (Supplementary Figure 8c-f). When Peak Force Tapping mode is used, no difference is apparent between the two magnifications (Supplementary Figure 8g,h), indicating that the tip-sample interactions are much reduced, and therefore that they have a lesser effect on the experimental measurements.

Supplementary Figures

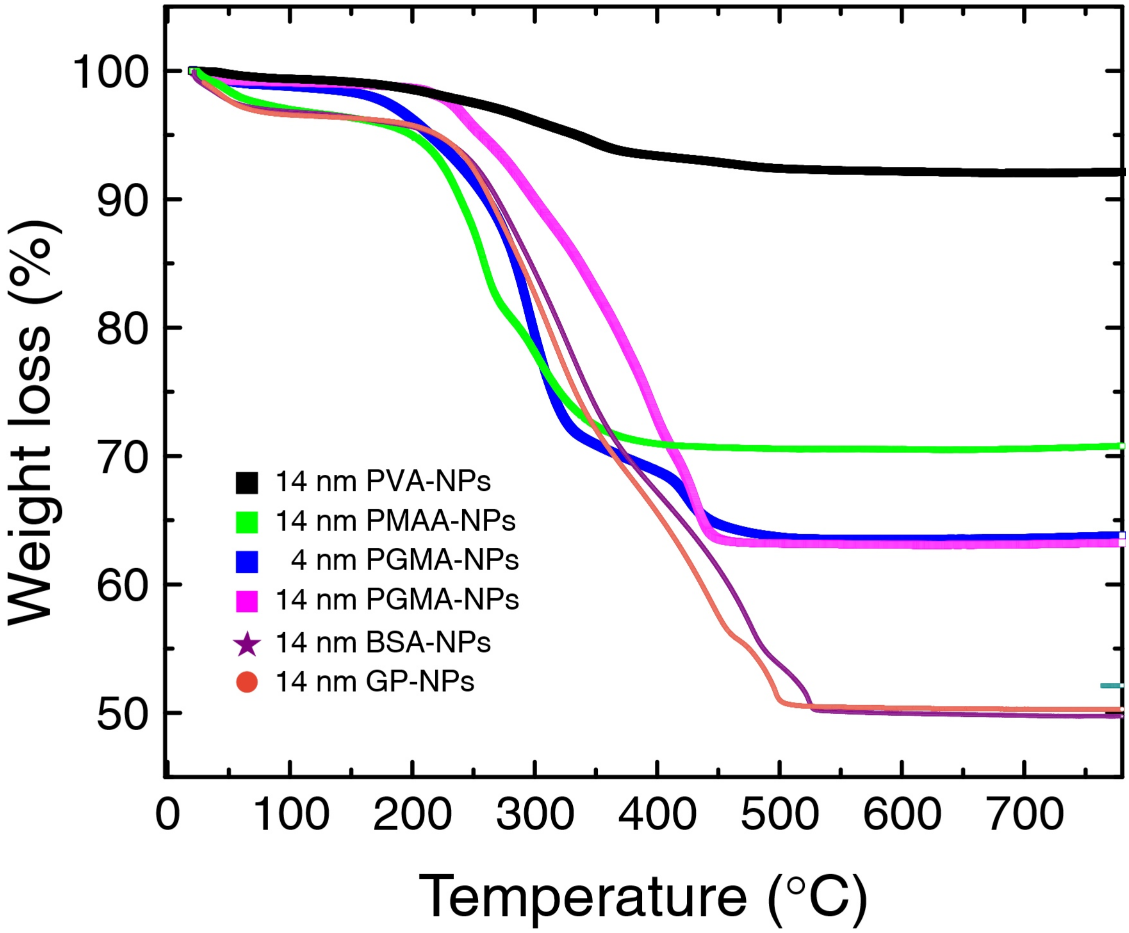

Supplementary Figure 1. Thermogravimetric analysis (TGA) of nanoparticles stabilised with proteins and polymers. The analysis was performed in air at a heating rate of 10°C/min. Water is removed up to 200 °C, and all proteins and polymers decomposed between 200 – 550 °C. All remaining residues correspond to gold. The compositions of the stabilised NPs are summarised in Supplementary Table 2.

a

b

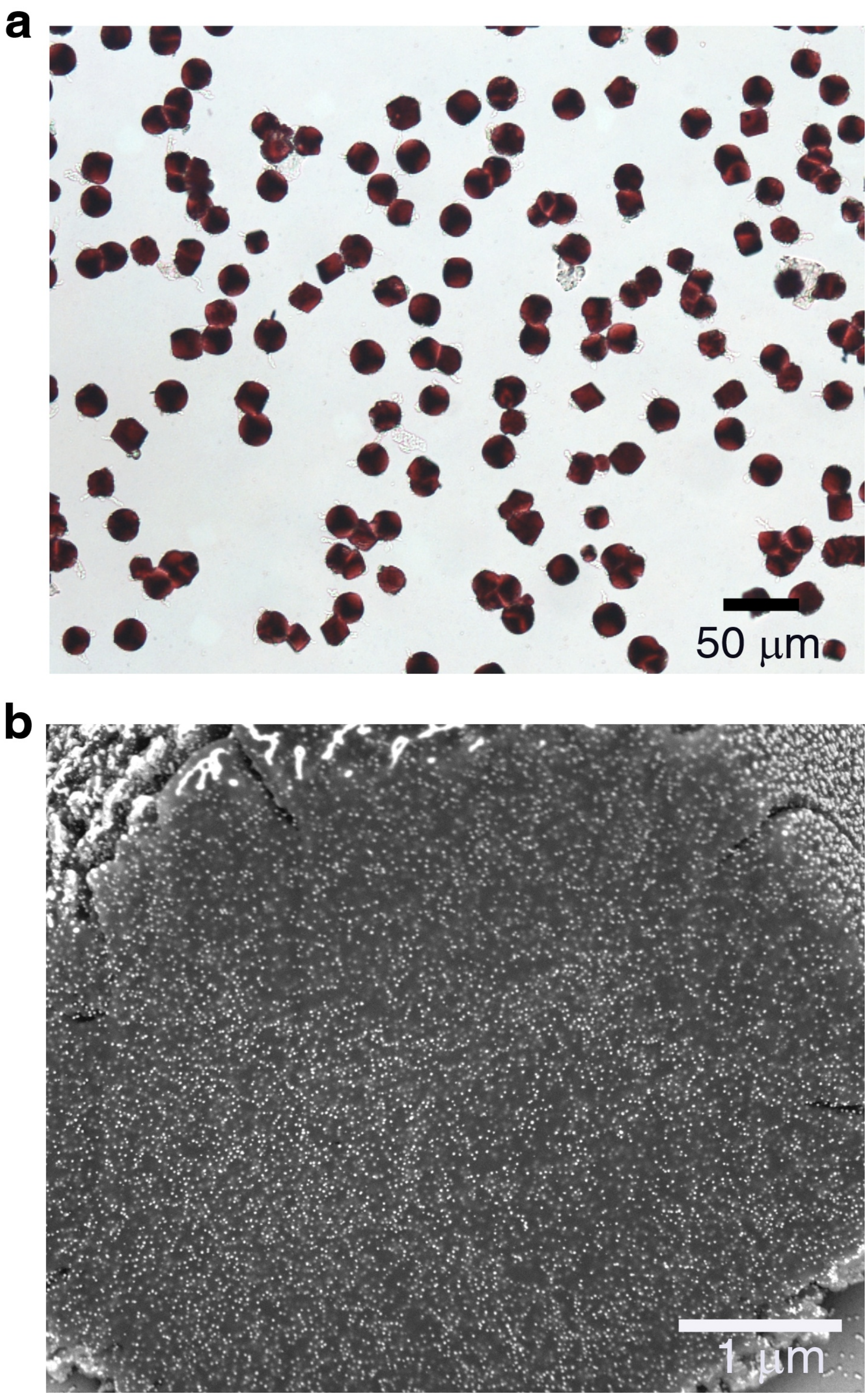

Supplementary Figure 2. Optical micrograph (a) and SEM image (b) of calcite crystals precipitated at [Ca] = 20 mM in the presence of 14 nm aminated BSA-NPs. The cross section shown in (b) was prepared using FIB.

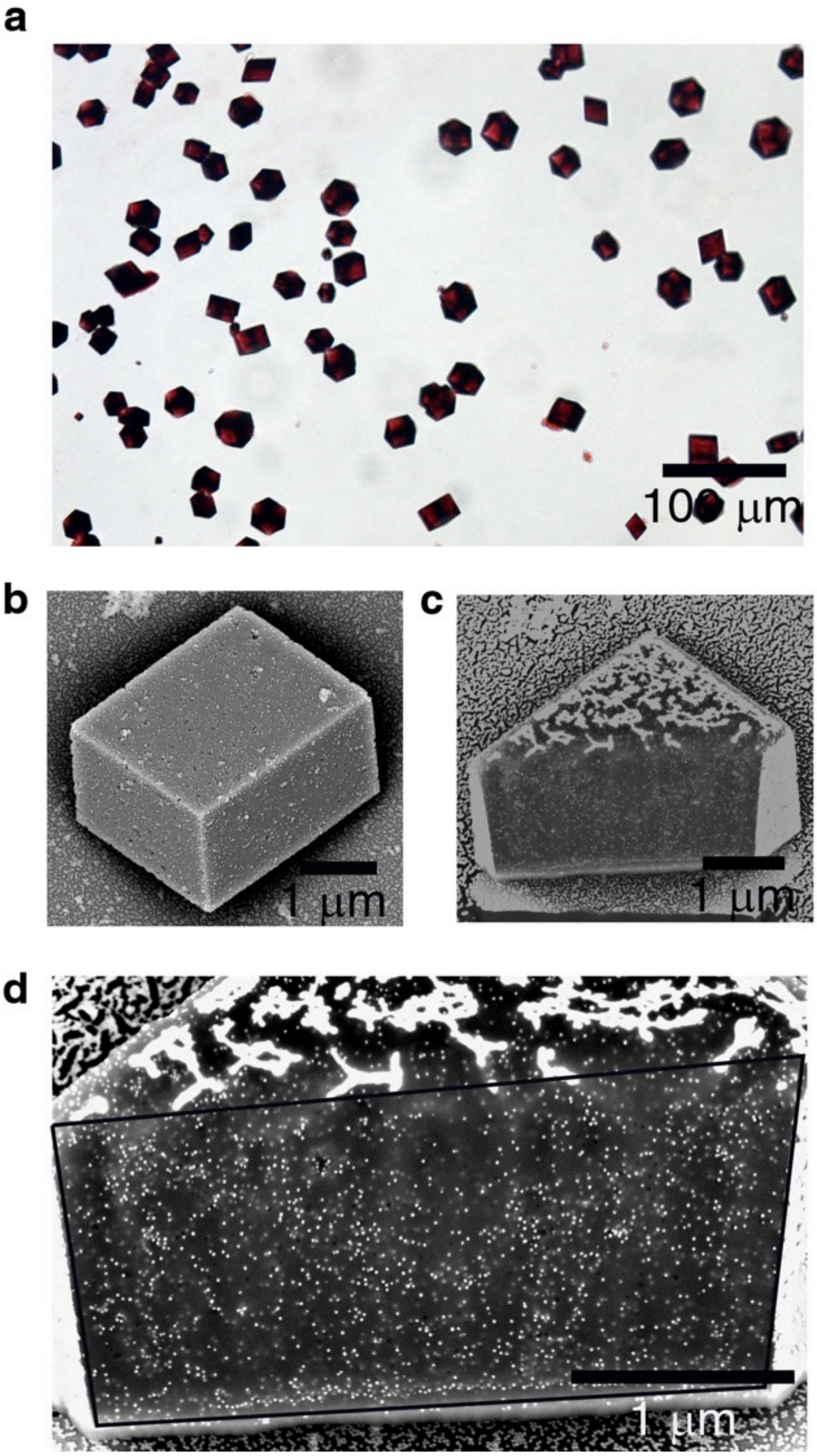

Supplementary Figure 3. Optical micrograph (a) and SEM images (b-d) of calcite crystals precipitated at [Ca] = 20 mM in the presence of 14 nm de-sialylated GP-NPs where the sialic acid terminal groups have been removed from the protein. The cross sections shown were prepared using FIB.

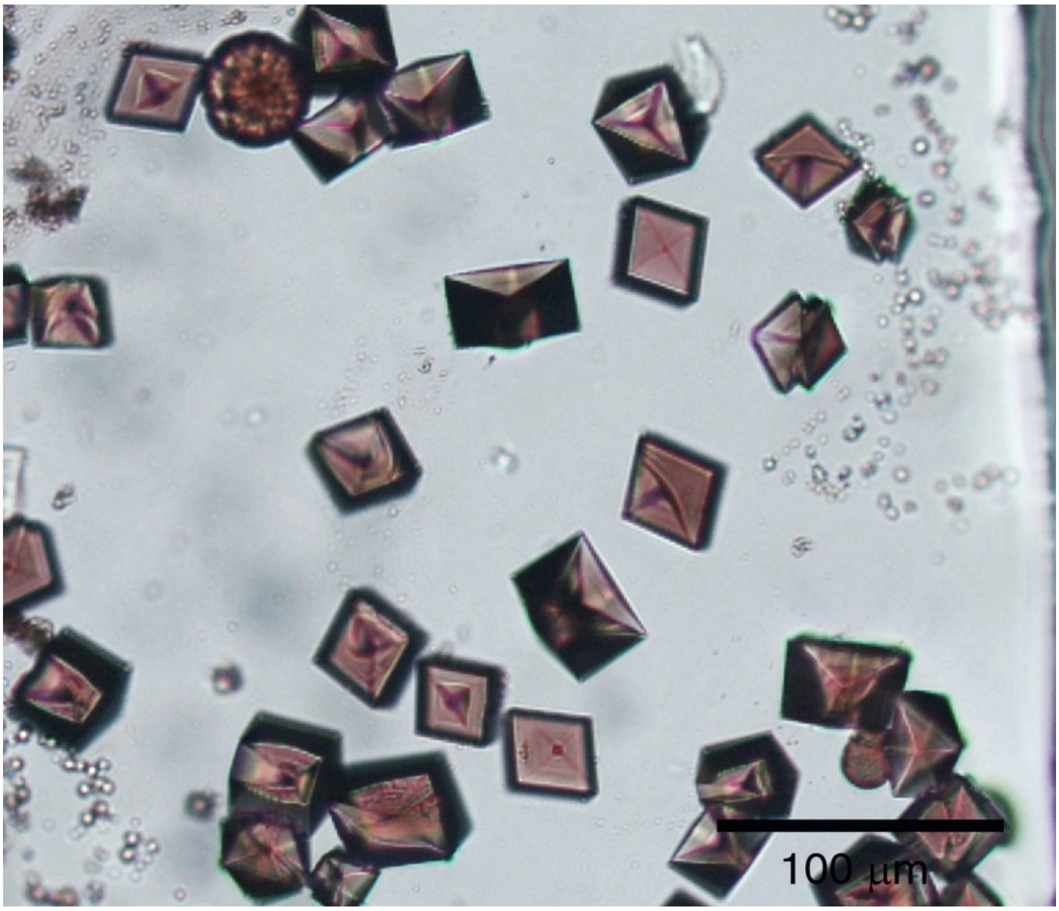

Supplementary Figure 4. Optical micrographs of calcite crystals precipitated at [Ca] = 20 mM in the presence of 14 nm PHEMA-NPs. PHEMA readily forms hydrogels in water and thus does not coat the nanoparticles well. This reduces the efficiency of NP occlusion within the crystals.

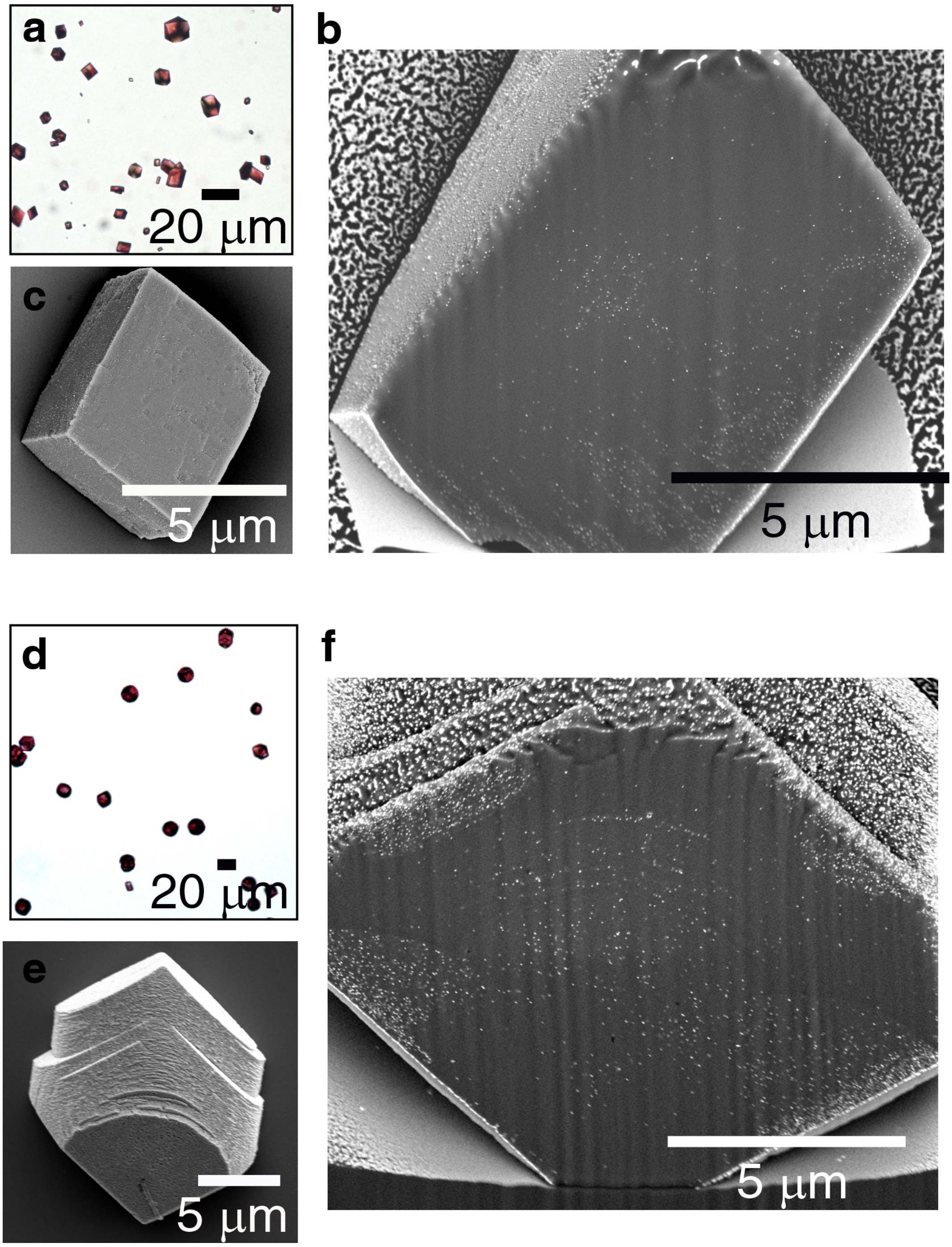

Supplementary Figure 5. Optical micrographs (a and d) and SEM images (b, c, e f) of calcite crystals precipitated at [Ca] = 1.5 mM in the presence of 0.01 wt% (a) 14 nm PGMA-NPs and (d) 14 nm PMAA-Au NPs. The elongated morphology of crystals in (d) is due to the preference of the NPs for the acute step/kinks.

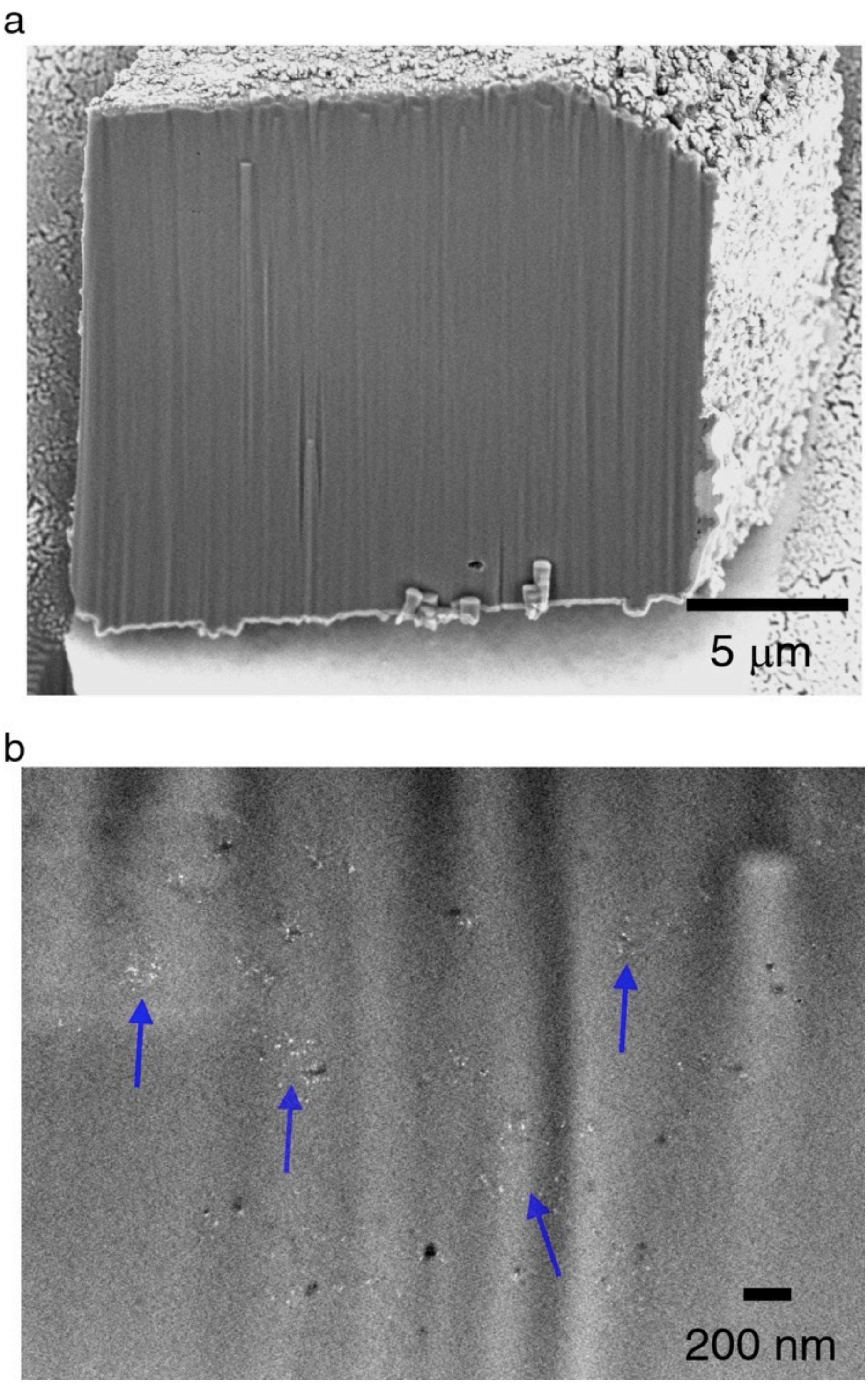

Supplementary Figure 6. (a-b) SEM images of FIB-sectioned calcite crystals precipitated at [Ca] = 20 mM in the presence of 0.05 wt% 5 nm PAH-NPs. (b) High magnification image of (a), where arrows indicate some small areas of occluded PAH-NPs. PAH interacts strongly with carbonate ions in the crystallisation solution, resulting in significant aggregation of the NPs, and therefore negligible occlusion.

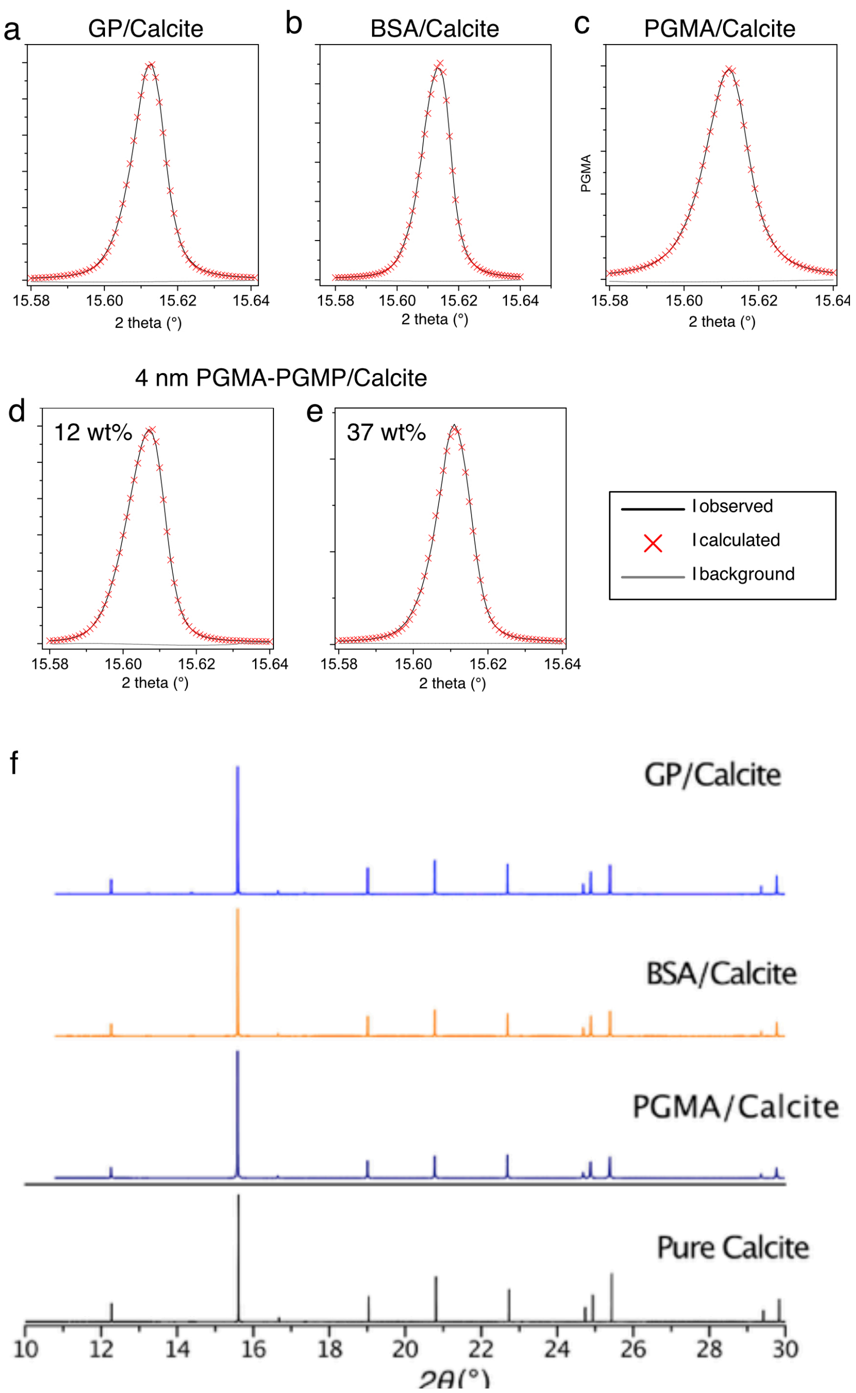

Supplementary Figure 7. (a-e) Line profile analyses of the {104} reflections of calcite nanocomposites. (f) High resolution powder x-ray diffractograms of calcite grown in the presence of proteins (BSA, GP) and PGMA alone.

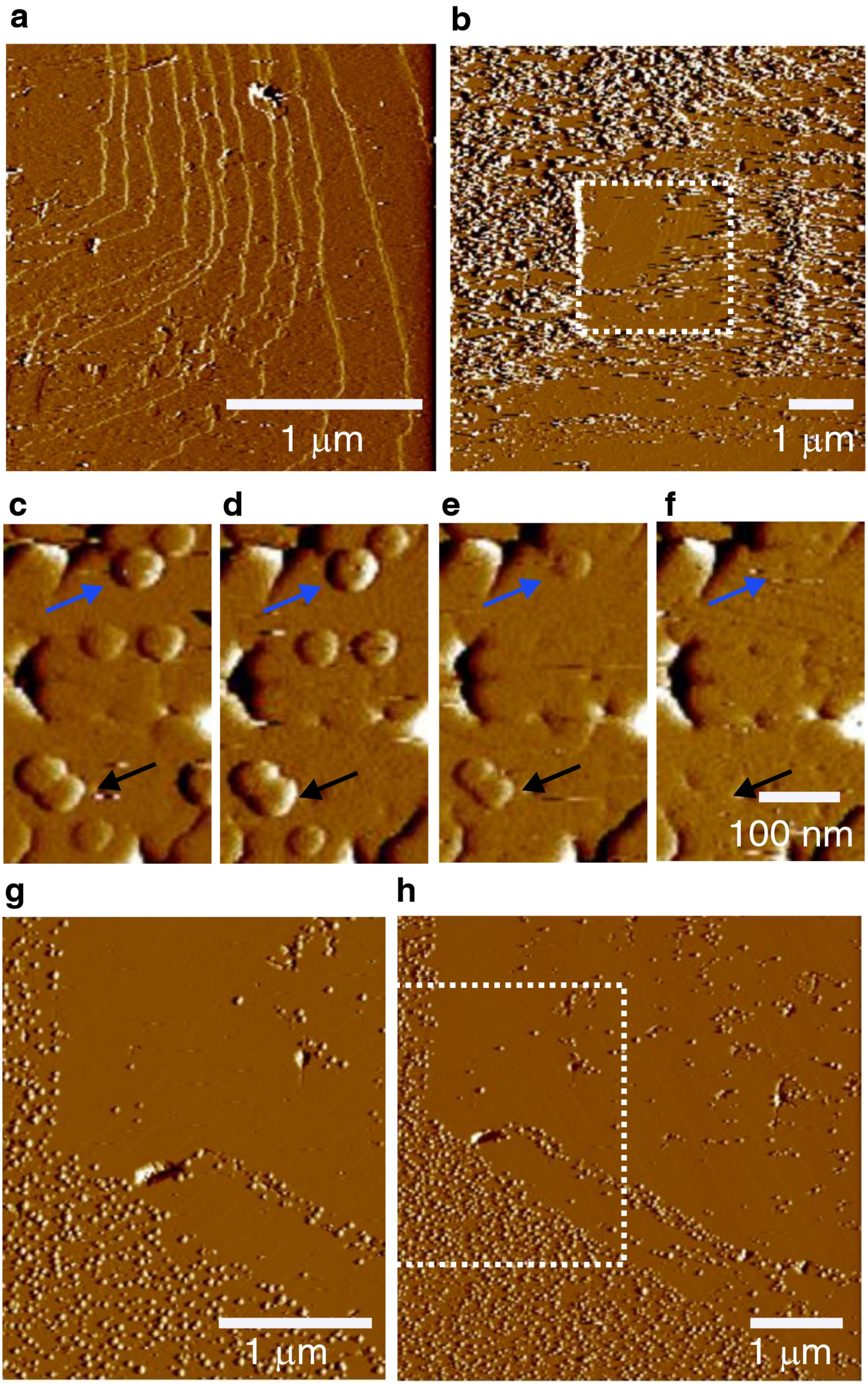

Supplementary Figure 8. Comparison of different AFM operating modes. AFM images of calcite crystals grown in the presence of 14 nm PGMA-NPs. (a, b) Deflection micrographs taken in contact

mode (with the contact force minimised) in the same area at two different magnifications. The low magnification micrograph (a) was obtained directly after the high magnification micrograph (b). The box in (b) shows the previous scan location. (c-f) Sequence of deflection images taken in contact mode, at 3 min intervals, showing incorporation of PGMA-NPs. During contact mode imaging, particles that were partially incorporated could be imaged, but no new particles were observed in the region of interest. The arrows highlight 2 individual NPs that can be seen to decrease in size over time, and ultimately become fully occluded. (g, h) Peak Force Error images taken in the same area at two different magnifications. The low magnification micrograph was obtained directly after the high magnification micrograph (3 min). The box in (h) shows the previous scan location.

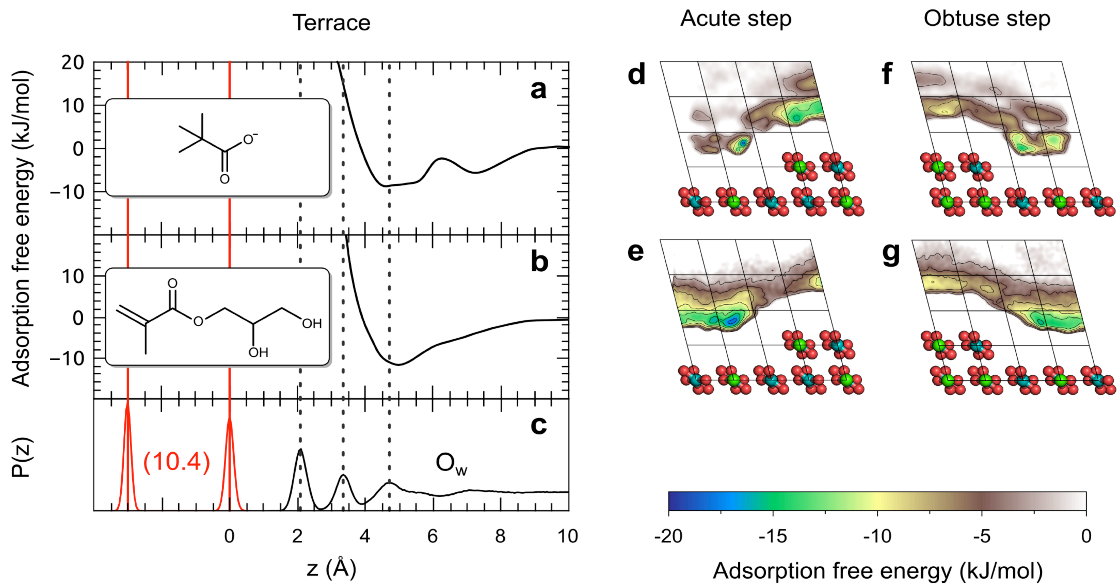

Supplementary Figure 9. The adsorption free energies for three organic molecules (shown in the insets) at terraces (a-b), the acute step (d,e) and the obtuse step (f.g). (c) shows the probability density along z for Ca ions in the top two calcite surfaces (red) and the water (black), with the position of the first three water layers shown for reference (dashed lines). The grids in (d-g) show the equilibrium lattice sites for calcite, and the contour lines correspond to 2.5 kJ/mol (1 kT at 300 K).

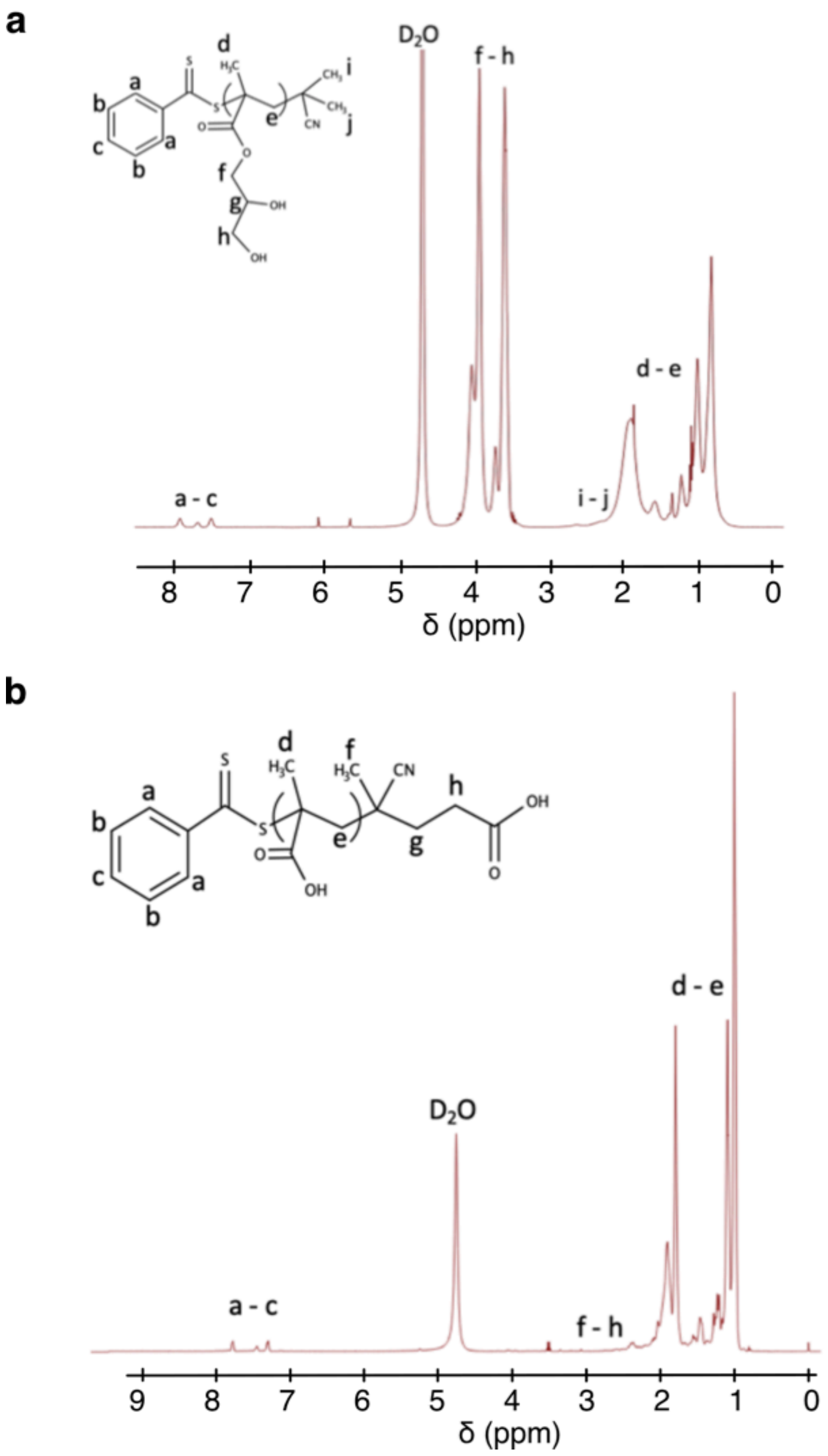

Supplementary Figure 10. ^{1}H NMR spectroscopy of the synthesised polymers (a) PGMA and (b) PMAA.

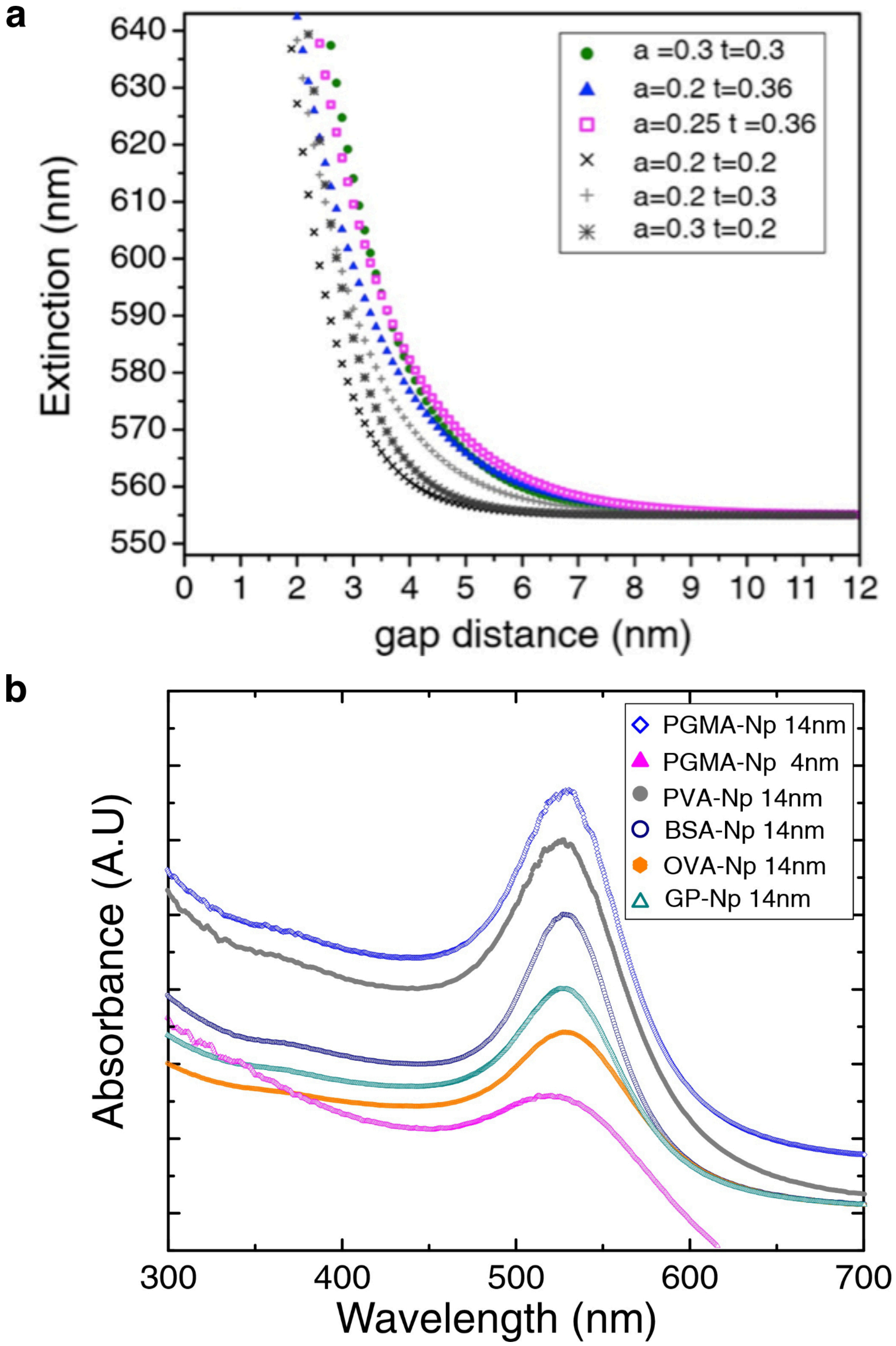

Supplementary Figure 11. (a) Extinction wavelength versus gap-distance estimated using the universal ruler equation. The decay constant τ and particle coupling strength (a) was varied to achieve the best fit; a = 0.25 and t = 0.36 gave the best fit to our experimental data. (b) UV-Vis spectra of protein- and polymer-stabilised NPs. No aggregation is observed.

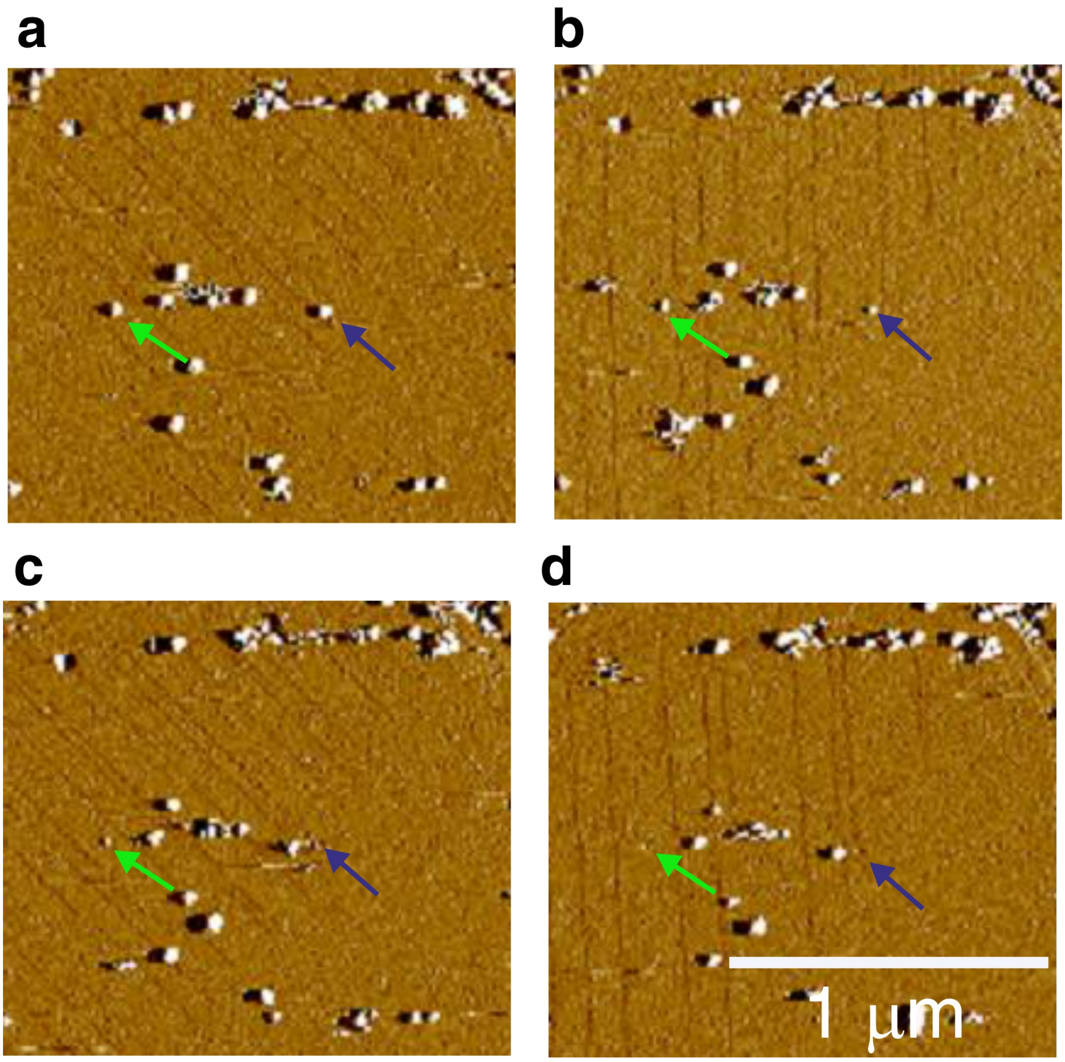

Supplementary Figure 12. AFM Peak Force Error images of calcite crystals grown *in situ* in the presence of 14 nm GP-NPs. (a-d) Images were taken at 4.5 min intervals. The green and blue arrows highlight individual particles during incorporation, where these can be seen to decrease in size and then become fully incorporated.

Supplementary Tables

Supplementary Table 1. Summary of the organic macromolecules used in this work. The sequences of proteins were found from NCBI and on SwissProt.

Protein/ polymer	Description	MW
**PGMA**	Synthetic OH<br>(diol motif)	10 k
**PVA**	Synthetic OH	10 k
**PMAA**	Synthetic COOH	10 k
**BSA**	***Serum protein***<br>Commonly used as a reference in several assays<br>99 acidic (17 %) and 82 basic (14 %) residues, 10 sites of physphorylation and 3 sites of lysine modifications.	66.5 kDa<br>583 residues and no carbohydrates<br>theoretical Pl 5.6
**Aminated BSA**	Chemically modified BSA	N/A
**GP**	***Glycoprotein from bovine plasma***<br>High levels of carbohydrates (45 %) of which 11 % are sialic acid groups.	40 kDa<br>183-187 residues.<br>Pl 2.8-3.8
**Desialylated GP**	Chemically modified BSA	N/A

Supplementary Table 2. Summary of characterisation of the polymer and protein stabilised nanoparticles.

	Composition from TGA analysis		Zeta Potential at pH = 9 and [Ca] = 20 mM (mV)	Z-average Diameter (nm)
	Au wt%	Polymer/protein wt%		
**4 nm PGMA-NP**	63	37	-3 (2)	16 (3)
**14 nm PGMA-NP**	63	37	-2 (2)	34 (1)
**14 nm PMAA-NP**	73	27	-40 (3) (at [Ca] = 1 mM)	32 (3)
**14 nm PVA-NP**	92	8	0 (1)	28 (3)
**14 nm GP-NP**	53	47	-6 (2)	36 (4)
**14 nm BSA-NP**	53	47	-7 (3)	32 (5)

Supplementary Table 3. Summary of the characterisation of the NP/calcite samples, where these were analysed using atomic absorption spectroscopy AAS). Standard deviation of AAS measurement was within 0.1 ~ 0.5 % for all the samples.

Nanocomposite Crystals	Protein/polymer Stabilised Au in Calcite (wt%)	Description
**14 nm PGMA-NP/ Calcite**	33 wt% by AAS<br>34 wt% by XRD<br>41 wt% from Tomography	ADM: [Ca] = 20 mM<br>NPs = 0.05 wt%
**14 nm PGMA-NP/ Calcite**	19	ADM: [Ca] = 20 mM<br>NPs = 0.01 wt%
**4 nm PGMA-NP/ Calcite**	26	ADM: [Ca] = 20 mM<br>NPs = 0.025 wt%
**4 nm PGMA-NP/ Calcite**	37	Kitano method<br>NPs = 0.02 wt%
**4 nm PGMA-NP/ Calcite**	12	Kitano method<br>NPs = 0.005 wt%
**4 nm PGMA-NP/ Calcite**	2.7	Kitano method<br>NPs = 0.001 wt%
**14 nm PVA-NP/ Calcite**	8	ADM: [Ca] = 20 mM<br>NPs = 0.05 wt%
**14 nm GP-NP/ Calcite**	13	
**14 nm Desilicated GP/ Calcite**	11	
**14 nm BSA-NP/ Calcite**	11	
**14 nm Aminated BSA-NP/ Calcite**	9	

Supplementary Table 4. Summary of lattice parameters and goodness of fit parameter is with SD obtained using high resolution synchrotron PXRD analysis (HR-PXRD). The lattice parameters and strain parameters were obtained using Rietveld analysis and line profile analysis respectively.

	**a=b lattice parameter**	**c lattice parameter**	**Goodness of Fit (line profile analysis) (104)**	**Goodness of Fit (rietveld analysis)**
**BSA/Calcite**	4.990606(5)	17.07053(25)	3.62	5.89
**GP/ Calcite**	4.990771(3)	17.07131(21)	3.31	7.07
**PGMA/ Calcite**	4.990637(7)	17.07325(35)	2.09	5.49
**4 nm PGMA-NP/ Calcite (12 wt%)**	4.989216 (4)	17.06909(29)	3.72	8.98
**4 nm PGMA-NP/ Calcite (37 wt%)**	4.990455(5)	17.07481(28)	4.68	7.00
**Pure Calcite**	4.990709(3)	17.06675(18)	2.05	3.64

Supplementary Videos

Supplementary Video 1:

Combined raw and reconstructed TEM tomography of a 14 nm GP-NP/calcite crystal.

Supplementary Video 2

Serial sectioning SEM using FIB milling through an entire calcite crystal containing 14 nm PGMA-NPs.

Supplementary Video 3

Combined raw and reconstructed STEM tomography of a 14 nm PGMA-NP/calcite crystal.

Supplementary Video 4

Raw STEM tilt series of a 4 nm PGMA-NP/calcite crystal.

Supplementary References

1. Ghosh SK, Pal T. Interparticle coupling effect on the surface plasmon resonance of gold nanoparticles: From theory to applications. *Chem Revs* **107,** 4797-4862 (2007).

2. Jain PK, Huang WY, El-Sayed MA. On the universal scaling behavior of the distance decay of plasmon coupling in metal nanoparticle pairs: A plasmon ruler equation. *Nano Letts* **7,** 2080-2088 (2007).

3. Hendley CT, Fielding LA, Jones ER, Ryan AJ, Armes SP, Estroff LA. Mechanistic insights into diblock copolymer nanoparticle-crystal interactions revealed via in situ atomic force microscopy. *J Am Chem Soc* **140,** 7936-7945 (2018).
