# Supplementary material for: Hydroxyl-rich macromolecules enable the bio-inspired synthesis of single crystal nanocomposites
Source: Nat Commun. 2019 Dec 12;10:5682. doi: 10.1038/s41467-019-13422-9 (PMC6908585; doi:10.1038/s41467-019-13422-9)
Supplement: Supplementary file 3 — Description of Additional Supplementary Files [file 41467_2019_13422_MOESM3_ESM.pdf]

Description of Additional Supplementary Files

File Name: Supplementary Movie 1
Description: Combined raw and reconstructed TEM tomography of a 14 nm GP-NP/calcite crystal.

File Name: Supplementary Movie 2
Description: Serial sectioning SEM using FIB milling through an entire calcite crystal containing 14 nm PGMA-NPs.

File Name: Supplementary Movie 3
Description: Combined raw and reconstructed STEM tomography of a 14 nm PGMA-NP/calcite crystal.

File Name: Supplementary Movie 4
Description: Raw STEM tilt series of a 4 nm PGMA-NP/calcite crystal.
